# Artificial gravity protects bone and prevents bone marrow adipose tissue accumulation in humans during 60 d of bed rest

**DOI:** 10.1093/jbmr/zjaf119

**Published:** 2025-08-28

**Authors:** Kathryn Culliton, Gerd Melkus, Adnan Sheikh, Tammy Liu, Alain Berthiaume, Gabi Armbrecht, Guy Trudel

**Affiliations:** Bone and Joint Research Laboratory, Inflammation and Chronic Disease Program, Ottawa Hospital Research Institute, Ottawa, ON, K1H 8L6, Canada; Department of Radiology, Radiation Oncology and Medical Physics, University of Ottawa, Ottawa, ON, K1H 8M5, Canada; Department of Radiology, University of British Columbia, Vancouver, BC, V6T 1Z2, Canada; Bone and Joint Research Laboratory, Inflammation and Chronic Disease Program, Ottawa Hospital Research Institute, Ottawa, ON, K1H 8L6, Canada; Department of Radiology, Radiation Oncology and Medical Physics, University of Ottawa, Ottawa, ON, K1H 8M5, Canada; Department of Radiology, Charité–Universitätsmedizin Berlin, Freie Universität Berlin and Humboldt-Universität zu Berlin, Berlin, 10117, Germany; Bone and Joint Research Laboratory, Inflammation and Chronic Disease Program, Ottawa Hospital Research Institute, Ottawa, ON, K1H 8L6, Canada; Department of Cellular and Molecular Medicine, University of Ottawa, Ottawa, ON, K1H 8M5, Canada; Division of Physical Medicine and Rehabilitation, Department of Medicine, The Ottawa Hospital, Ottawa, ON, K1H 8M2, Canada

**Keywords:** bone marrow adipose tissue, inactivity, bone mineral density, artificial gravity

## Abstract

Inactivity has been associated with increased bone marrow adipose tissue (BMAT) and bone loss. Artificial gravity (AG) may prevent these complications. This randomized controlled trial investigated the effectiveness of AG at 2 g at the feet to prevent lumbar vertebral BMAT accumulation and bone loss. Twenty-four participants (16 male, 8 female) were bedridden for 60 d at 6° head down tilt. They were randomly assigned to bedrest only (*n* = 8), continuous supine centrifugation (cAG; 30 min/d), or intermittent supine centrifugation (iAG; 6 bouts of 5 min/d). Serial 3T magnetic resonance (MR) measured BMAT while DXA measured BMD in the lumbar vertebrae before, during, and after bedrest. After 60 d of bedrest, vertebral BMAT was higher in controls, +3.93% (95% CI: −0.28 to 8.14), compared to cAG and iAG interventions. After 60 d of bedrest, male controls BMAT increased 5.81% (95% CI: 2.01 to 9.61) compared to −1.35% (95% CI: −5.74 to 3.04) and 1.23% (95% CI: −1.53 to 3.99) for male cAG and iAG participants, respectively. This difference between interventions was significant: *X*^2^(2) = 8.487, *p* = .014. In addition, while control male participants showed decreased BMD after 60 d of bedrest (−0.02 g/cm^2^; 95% CI: −0.05 to 0.00), the male participants receiving iAG showed no decrease in BMD during bedrest (0.00 g/cm^2^; 95% CI: −0.04 to 0.05). The modulation of BMAT was inversely correlated with BMD at the same vertebrae. Recreating an axial force vector mechanically on horizontalized participants prevented BMAT accumulation and demineralization. These findings suggest exploring technological advances to translate these clinical benefits to populations at risk of acute or chronic bone loss.

## Introduction

Inactivity and skeletal offloading have deleterious effects on most organs, including bones.^1–4^ Inactivity in the form of bed rest may be unavoidable when treating acute illnesses or associated with limited mobility in the chronically ill. Although not inactive, astronauts also undergo prolonged periods of skeletal offloading in microgravity environments.

The bone marrow and bone are sensitive to inactivity, loading, and exercise.^5–8^ The bone marrow undergoes a progressive conversion towards increase adipose content over the course of an individual’s lifetime.^9–11^ This conversion, where the red hemopoietic marrow is replaced with fat can be accelerated by numerous factors including inactivity.^12–18^ Several studies reported bone marrow adipose tissue (BMAT) modulation during bed rest at the lumbar vertebrae.^5^^,^^6^^,^^12^ Bed rest, specifically head-down-tilt (HDT) bed rest, is a ground-based microgravity analogue as it mimics the skeletal offloading, fluid redistribution, bone, muscle, and cardiovascular adaptations to space. Index studies suggested BMAT increases during HDT bedrest,^5^^,^^6^ dependent on the duration and type of countermeasure being tested.^12^^,^^13^ In the days to months after bed rest, downregulation of BMAT signal has been reported.^12^ Decreased BMAT during rehabilitation was also measured on 14 astronauts reambulating after 6-mo missions on the international space station, confirming the value of the model.^12^^,^^14^ The conversion and reconversion of bone marrow can be measured precisely using non-invasive magnetic resonance (MR) imaging.^15^^,^^16^ Countermeasures to overcome the effects of bedrest on BMAT and bone have been studied. In a clinical trial, 24 female participants were submitted to 60 d of bedrest. Eight participants tested a resistive and aerobic exercise regimen, eight participants tested a leucin enriched diet, and eight participants had no countermeasure. The vertebral BMAT content increased 2.5% (on a BMAT scale of 0-100) during HDT bed rest, and the increase persisted after 1 yr of reambulation; there was no effect of the countermeasures.^5^ A similar trial (60-d bed rest) was conducted in male participants, where 8 participants received resistive exercise, 8 participants received resistive exercises supplemented with whole-body vibration, and 8 participants had no countermeasure. In the control group, vertebral BMAT content increased 3.3 percentage points and both countermeasures successfully prevented BMAT accrual during bed rest.^6^ These studies provided the first experimental evidence in humans that bed rest induced fat accumulation in the vertebral bone marrow; that could be prevented by mechanical countermeasures. A subsequent cross over study of 10 patients submitted to a much shorter 20 d of HDT bed rest with half receiving a high-protein diet intervention measured no significant change in vertebral BMAT.^13^ A recent study of 24 male participants submitted to 60 d of HDT bedrest, where half received a nutritional intervention measured no BMAT changes during the HDT bedrest, a significant BMAT downregulation by 10 percentage points at 30 d of reambulation and no effect of the diet supplements. BMAT accumulation is also associated with loss of BMD.^5^^,^^17–21^ Most studies on the effect of inactivity on BMAT recruited men.^5^^,^^6^^,^^13^^,^^14^ However, consistent differences in the level and modulation of BMAT have been reported compared to women.^5^^,^^14^ Therefore, direct sex-based analysis of BMAT modulation with inactivity are needed. The inverse relationship between BMAT and BMD has long been reported in men, women, with aging and with osteoporosis.^18–21^ Decreased BMD has been consistently reported with bed rest^22–24^ and spaceflight.^25^^,^^26^ Many of the studies that have investigated BMAT accumulation have also reported BMD, and resistive exercises have prevented both BMAT accumulation and bones loss in people subjected to bed rest.^6^^,^^12^^,^^22–24^^,^^27^

In clinical situations where physical exercise or weightbearing cannot be implemented, such as an athlete during injury, or patients with paralysis, such as a paraplegia, or astronauts in microgravity, alternative physical countermeasures are required. Artificial gravity (AG) is of high interest to counter the effects of inactivity. Artificial gravity can be achieved through centrifugation by spinning an individual about an axis and 2G human short arm centrifuges have been tested.^26^^,^^27^ Short (5 d) and mid-duration (21 d) bed rest studies have demonstrated that AG preserved muscle size and neuromuscular function.^28^^,^^29^ Several AG protocols have been tested to maximize effectiveness while optimizing tolerability.

The objectives of this study were to: (1) test the effectiveness of two different AG protocols, continuous vs intermittent, on the lumbar vertebral BMAT and BMD during and after 60 d of HDT bed rest; (2) investigate a sex-specific BMAT modulation; (3) correlate the BMAT changes with BMD at the same lumbar vertebrae**.** Bedrest standard measures technical data were published.^30^ To our knowledge, the effect of AG on BMAT modulation and its correlation with BMD has never been reported.

## Methods

### Study design

In this prospective randomized clinical trial, 24 healthy volunteers participated in a 60-d, 6° HDT bed rest study. The study was conducted at the envihab facility of the German Aerospace Center (DLR) in Cologne, Germany. Participants completed the study in two consecutive campaigns (*n* = 12 each). In both campaigns, the participants were required to stay at the facility for 88 d: 14 d of baseline data collection (BDC), 60 d of bed rest, and 14 d of reambulation. Serial 3T MR scanning imaged the lumbar spine at 12 d before bed rest, after 31 and 60 d of bed rest, and at 8, 90, and 480 d of reambulation. The clinical trial was approved by the Ärztekammer Nordrhein (#2018143), the Ottawa Health Science Network Research Ethics Board (#20190023-01H), and the Federal Office for Radiation Protection (Z5-22464/2018-074-R-G). The study was prospectively registered at the German Clinical Trials Register (DRKS00015677).

### Artificial gravity intervention

Artificial gravity elicited via daily short arm centrifugation (radius 3.8 m) was used as a countermeasure to bed rest with participants randomly assigned to one of three experimental groups (*n* = 8 per group): non-centrifuge controls, continuous supine centrifugation (cAG; 30 min per day), or intermittent supine centrifugation (iAG; 6 bouts of 5 min per day). For the participants that were subjected to daily centrifugation, their position with respect to the center of rotation as well as the rotational speed of the centrifuge was adjusted to achieve an acceleration of 1 g at the estimated center of mass and 2 g at the feet. Centrifugation was carried out in the horizontal position (ie, 0° HDT) and achieved with the DLR short arm human centrifuge, available on https://www.nasa.gov/sites/default/files/atoms/files/m1_shortarmhumancentrifuge_updated.pdf.

### Magnetic resonance imaging protocols

Magnetic resonance imaging was performed at 3T (Biograph mMR, Siemens Healthineers) using the table built-in spine coil receiver array. The following sequences were used on all participants:

1. Sagittal 2D multislice proton-density weighted turbo spin-echo with (PDFS) and without (PD) fat saturation covering L2-L5 (repetition time = 2000 ms; echo time = 12 ms; FOV = 250 × 250 mm^2^; matrix = 448 × 224; slice thickness = 6 mm; echo train length = 3; number of averages = 1).
2. Sagittal 3D gradient echo 2-point Dixon sequence covering L2-L5 with fat- and water-only image reconstruction (repetition time = 5.77 ms; echo time (in phase) = 2.46 ms; echo time (out of phase) = 3.69 ms; FOV = 250 × 250 mm^2^; matrix = 256 × 256; slice thickness = 5 mm; flip angle = 10°; number of averages = 3; echo train length = 2).
3. Point RESolved Spectroscopy sequence of the L3, L4, and L5 vertebrae (repetition time = 10 s, echo time = 30 ms, number of averages = 4, voxel size = 15 × 15 × 15 mm^3^, receiver bandwidth = 2000 Hz, spectral points = 1024).

These protocols have been used for similar studies.^6–8^^,^^12–14^ Fat saturation and chemical shift sequences have shown reproducibility to measure fat fraction and correlation with fat content and histological findings.^31^^,^^32^

### Vertebral bone marrow adipose tissue

Three sagittal interpedicular slices from vertebral bodies L2-L5 were selected and polygonal regions of interest (ROI) were drawn manually over each vertebra. The superior and inferior endplates as well as the anterior and posterior cortices were excluded from the ROI. For proton density and Dixon acquisitions, ROIs were processed using an in-house, custom-written algorithm developed in MATLAB (MATLAB 2014a; Mathworks). The corresponding BMAT maps were:


$$ {\mathrm{BMAT}}_{\mathrm{PD}}=\frac{{\mathrm{Image}}_{\mathrm{PD}}-{\mathrm{Image}}_{\mathrm{PD}\mathrm{FS}}}{{\mathrm{Image}}_{\mathrm{PD}}} $$

$$ {\mathrm{BMAT}}_{\mathrm{DIXON}}=\frac{{\mathrm{Image}}_{\mathrm{fat}}}{{\mathrm{Image}}_{\mathrm{fat}}+{\mathrm{Image}}_{\mathrm{water}}} $$

where Image_PD_ and Image_PDFS_ are the images obtained without and with fat saturation, respectively; and Image_fat_ and Image_water_ are the images obtained with the Dixon technique above, see “Vertebral Bone Marrow Adipose Tissue.” The MR spectroscopy was processed using LCModel (Version 6.3-1L). Lipid peak areas at 0.9, 1.3, and 1.6 ppm were summed to represent the fat signal (S_fat_), and the water peak at 4.7 ppm represented the water signal (S_water_).


$$ {\mathrm{BMAT}}_{\mathrm{MRS}}=\frac{S_{\mathrm{fat}}}{S_{\mathrm{fat}}+{S}_{\mathrm{water}}} $$

### Bone mineral density

Bone mineral density of the lumbar spine (L1-L4) was measured using DXA with a Prodigy Full Pro system (GE Healthcare GmbH, manufacturer’s enCORE software (version 16.10.151)). The measurements were taken at several key time points: 12 d before bed rest (baseline), after 30 and 60 d of bed rest, and at 11, 420, and 720 d of reambulation. T-scores were calculated for L1-L4 using NHANES reference population data (20-30 yr of age) combined with Lunar reference population data (20-40 yr of age).

### Data and statistical analysis

The primary outcome was the vertebral fat fraction as a surrogate measure for vertebral BMAT. For PD and Dixon techniques, mean BMAT was determined by the average of three interpedicular slices of each vertebra L2-L5 on a scale of 0-100. For MRS, BMAT was averaged for L3-L5 and presented on the same scale of 0-100. For the primary outcome measure, mean change from baseline with 95% CIs are presented. All data are expressed as a change in raw BMAT on a scale of 0-100 and not a relative percentage change. Paired *t*-tests were used to assess differences between baseline and experimental time points with no correction for multiple hypothesis testing. The effect of sex (women, man) was tested for all timepoints using a Mann–Whitney U test. The effect of intervention (control, cAG, iAG) for all timepoints on BMAT and BMD was determined using a Kruskal–Wallis test.

## Results

**Figure 1:**
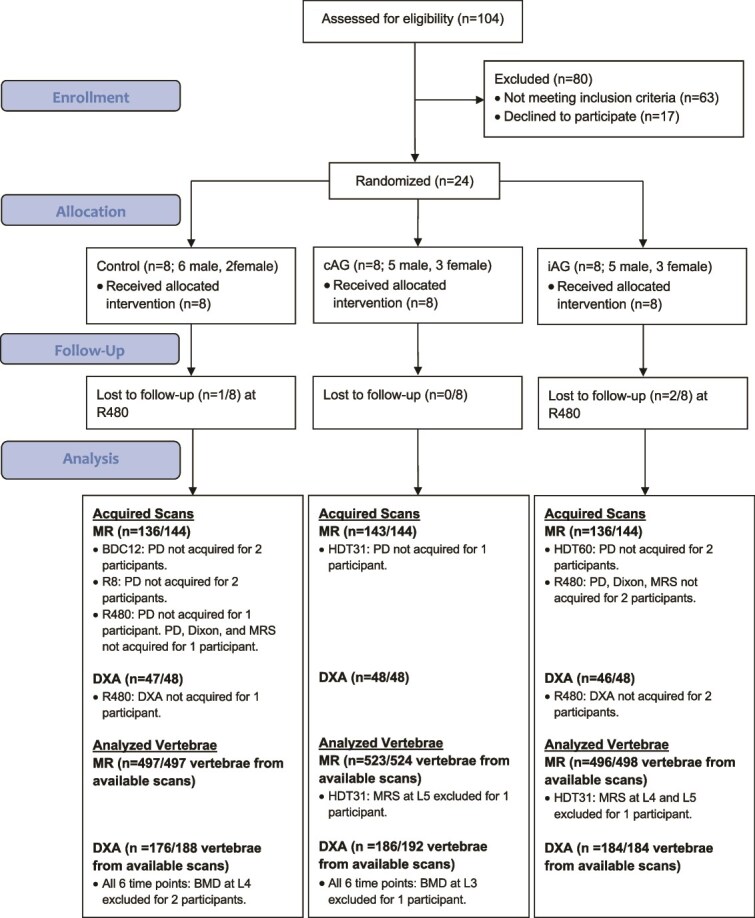
CONSORT chart. Twenty-four participants were randomized to three interventions (control, continuous artificial gravity (cAG), and intermittent artificial gravity (iAG)). The lumbar spine was imaged at 6 timepoints: BDC 12 d before bedrest (BDC12), head-down tilt bed rest days 31 and 60 (HDT31, HDT60), and Reambulation days 8, 90, and 480 (R8, R90, R480) using three MR quantitative techniques: proton density (PD), chemical shift (Dixon), and magnetic resonance spectroscopy (MRS). Dual energy X-ray absorptiometry (DXA) was performed 12 d before bed rest (BDC12), after 30 and 60 d of bed rest, and at 11, 420, and 720 d of reambulation. Three participants did not return for imaging at R480 due to the COVID-19 pandemic and in 7 participants, the scanner selected the wrong antenna for PD imaging. This resulted in 415 of a possible 432 MR, and 141 of a possible 144 BMD scans available for analysis. On each MR scan, bone marrow adipose tissue (BMAT) was measured at vertebrae L2, L3, L4, and L5 using PD and Dixon, and L3, L4, and L5 using MRS. three vertebrae on MRS scans were excluded from two participants at HDT31 due to artifact. Therefore, 1516 out of 1584 available vertebrae were analyzed for BMAT. BMD was measured by DXA at vertebral levels L1-L4. In 2 control participants, BMD at L4 was excluded, one due to a T-score < 1 SD, and the other due a T-score > 1 SD of the other vertebrae. In one participant from the cAG group, BMD at L3 was excluded due to a T-score > 1 SD than the other vertebrae. Therefore, 546 out of 576 available vertebrae were analyzed for BMD.

Eight women and 16 men were recruited and completed 60 d of HDT bedrest and early reambulation phases (R) of the study (Figure 1, Table 1). Three participants (two iAG, one control) did not return for follow up at R480. Technical issues led to individual vertebrae exclusions on MR and DXA imaging (Figure 1). As a result, BMAT was analyzed in 1516 out of a possible 1584 vertebrae and BMD was analyzed in 546 out of a possible 576 vertebrae (Figure 1). The three MR quantitative techniques for BMAT showed high correlations (PD-Dixon *r*^2^ = 0.89; PD-MRS *r*^2^ = 0.77; and Dixon-MRS *r*^2^ = 0.81; Figure S1).

**Table 1:** Summary of participant demographics recorded upon admission.

	**All**	**Control**	**cAG**	**iAG**	***p* value**
**Sex**	16 m; 8f	6 m; 2f	5 m; 3f	5 m; 3f	
**Age -years (95% CI)**	33.3 (29.4-37.2)	34.1 (27.4–40.8)	31.8 (23.6-40.0)	33.8 (24.7-42.9)	0.706
**BMI (95% CI)**	24.4 (23.5-25.3)	25.8 (24.1-27.5)	24.2 (22.6-25.8)	23.2 (21.9-24.5)	0.043
**Baseline BMAT (95% CI)**	44.1 (40.6-47.5)	41.7 (34.1-49.4)	48.2 (43.3-53.0)	42.4 (35.2-49.6)	0.365
**Baseline BMD (95% CI)**	1.20 (1.14–1.26)	1.26 (1.10-1.42)	1.18 (1.07-1.29)	1.16 (1.07-1.25)	0.498

Abbreviations: BMAT, bone marrow adipose tissue; cAG, continuous artificial gravity; iAG, intermittent artificial gravity.

### Effect of artificial gravity interventions on vertebral bone marrow adipose tissue

After 60 d of bed rest, vertebral BMAT was higher in control participants, +3.93% (95% CI: −0.28 to 8.14), compared to participants subjected to cAG and iAG interventions (*X*^2^(2) = 6.860, *p* = .032; Figure 3A). After 60 d of bed rest, participants subjected to cAG had an average change in BMAT of −1.21% (95% CI: −4.01 to 1.59), while participants subjected to the iAG intervention showed no change in BMAT, 0.00% (95% CI: −2.48 to 2.48). While there were differences between groups after 60 d of bedrest, no group was significantly different from their baseline BMAT measure.

At reambulation day 8, participants who received the cAG or the iAG interventions showed significantly lower BMAT compared to their baseline values: cAG: −5.04 (95% CI: −8.63 to −1.45) and iAG −2.95 (95% CI: −4.47 to −1.43); this was also observed at R90: cAG: −6.28 (95% CI: −11.01 to −1.55) and iAG: −4.11 (95% CI: −5.61 to 2.60; Figure 4A). Control participants showed no significant decrease in BMAT at reambulation compared to baseline. There were no significant differences between groups. BMAT was comparable to baseline after 480 d of reambulation in all three groups (Figure 4A).

### Sex-based modulation of vertebral bone marrow adipose tissue

At baseline BMAT was lower in female compared to male participants, 40.17% (95% CI: 34.72-45.63) vs 45.81% (95% CI: 41.19-50.44), respectively, for a M:F physiological difference of 5.64% (Figure 4A). This difference was significant, *U* = 31, *p* = .043. After 60 d of HDT bed rest, the M:F difference was accentuated to 9.58% explained by male participants BMAT +2.14% (95% CI: −0.17 to 4.45) and female participants BMAT −1.56% (95% CI: −4.36 to 1.24) compared to their respective baseline values (Figure 4B). Both sexes showed a downregulation of BMAT after 8 d of reambulation. Female participants decreased by −4.14% (95% CI: −5.80 to −2.48) and male participants decreased by −2.35% (95% CI: −4.56 to −0.14) compared to their respective baselines with no significant differences between sexes (*U* = 36, *p* = .086; Figure 3B). After 90 d of reambulation, participants of both sexes still showed significant decreases in BMAT compared to baseline, −3.24% (95% CI: −5.80 to −0.68) and −4.98% (95% CI: −7.45 to −2.51) for female and male participants, respectively (Figure 3B) with no significant differences between sexes. The BMAT after 480 d of reambulation was not significantly different from baseline for either group. The 5.41% M:F difference after 480 d of reambulation was no longer statistically significant (Figure 4A).

### Sex-based response of vertebral bone marrow adipose tissue to bed rest and to artificial gravity

Only male control participants showed increased BMAT with bed rest compared to baseline. After 60 d of bed rest, male control participant BMAT increased 5.81% (95% CI: 2.01 to 9.61) compared to −1.35% (95% CI: −5.74 to 3.04) and 1.23% (95% CI: −1.53 to 3.99) for male cAG and iAG participants, respectively (Figure 3C). This difference between interventions was significant: *X*^2^(2) = 8.487, *p* = .014. Although female participants featured no significant differences between interventions at any study time point, the low number of female participants (*n* = 2-3/group) precludes further conclusions at this time (Figure 3D).

### Effect of bed rest and reambulation on vertebral bone marrow adipose tissue

**Figure 2:**
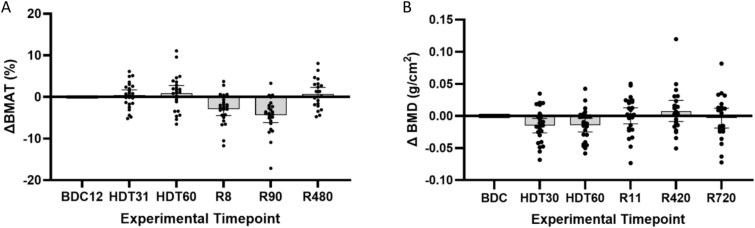
(A and B) Change in lumbar spine bone marrow adipose tissue and BMD with bedrest. (A) Mean changes in BMAT from baseline of the lumbar spine for all participants combining PD, Dixon, and MRS quantitative MR data. Reambulation after 60 d of bedrest significantly decreased BMAT. (B) Mean change in BMD from baseline of the lumbar spine for all participants. Bed rest significantly decreased lumbar vertebrae BMD. Whiskers correspond to 95% CIs. BMAT, bone marrow adipose tissue; PD, proton density; MRS, MR spectroscopy; BDC, baseline data collection; HDT, head-down tilt bedrest; R, reambulation; cAG, continuous artificial gravity; iAG, intermittent artificial gravity. *p < .05 compared to baseline.

At baseline, mean lumbar vertebrae BMAT was 44.1% (95% CI: 40.6 to 47.5) for all participants across sex, vertebral levels, interventions, and MR techniques. Bone marrow adipose tissue was +0.91% (95% CI: −0.92 to 2.74) after 60 d of bedrest, this change was not statistically significant (*t*(23) = −0.780, *p* = .443, Figure 2A). Ambulation after bed rest decreased lumbar vertebral BMAT (Figure S2). At reambulation day 8, vertebral BMAT had decreased −2.95% (95% CI: −4.48 to −1.41) for all participants compared to baseline (*t*(23) = 3.831, *p* = .001, Figure 2A). After 90 d of reambulation, BMAT had further decreased to −4.40% (95% CI: −6.17 to −2.63) below baseline (*t*(23) = 5.089, *p* < .001, Figure 2A). Bone marrow adipose tissue was not statistically different than baseline after 480 d of reambulation. Stratification by MR technique is shown in Table S1 and Figure S2.

**Figure 3:**
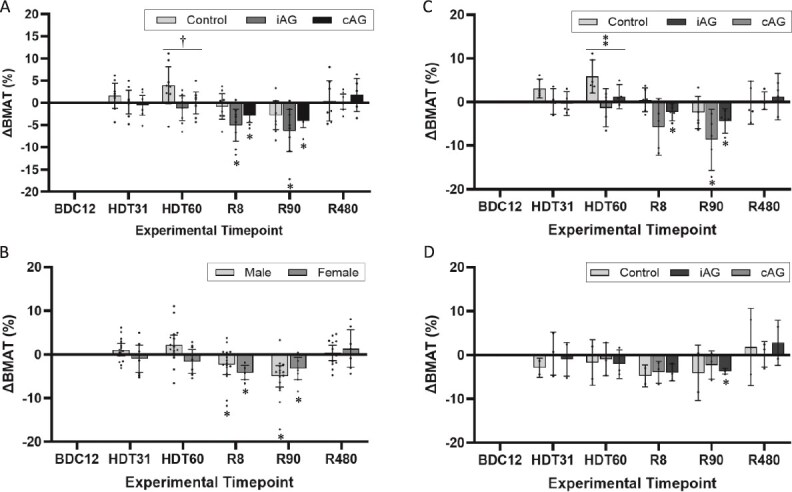
(A-D) Change in bone marrow adipose tissue stratified by intervention and sex. (A) Mean changes in BMAT of the lumbar spine from baseline stratified by intervention for all participants combining PD, Dixon, and MRS quantitative data. The 2 AG intervention groups had significantly lower BMAT than controls after 60 d of bedrest. Both AG intervention groups showed significantly decreased BMAT in the first 3 mo of reambulation. (B) Mean change in BMAT from baseline of the lumbar spine stratified by sex for all participants combining PD, Dixon, and MRS quantitative MR data. Both female and male participants significantly decreased their BMAT in the first 3 mo of reambulation after 60 d of bedrest. Mean change in BMAT from baseline of the lumbar spine stratified by intervention for male (C) and female (D) participants averaging PD, Dixon, and MRS quantitative data. Both AG intervention groups had significantly lower BMAT than controls after 60 d of bedrest in male but not in female participants. The iAG group showed decreased BMAT after 8 and 90 d of reambulation in male and after 90 d of reambulation in female participants, compared to baseline. Whiskers correspond to 95% CIs except for (D), where whiskers correspond to ±1 SD. SD is used for female participants due to the large 95% CI associated with *n* = 2, *n* = 3, and *n* = 3 for control, cAG, and iAG subgroups, respectively. BMAT, bone marrow adipose tissue; PD, proton density; MRS, MR spectroscopy; BDC, baseline data collection; HDT, head-down tilt bedrest; R, reambulation; cAG, continuous artificial gravity; iAG, intermittent artificial gravity. *p < .05 compared to baseline. ^†^*X*^2^(2) = 6.860, *p* = .032 comparing control, cAG, and iAG participants. ^$\overset{*}{{*}} $^*X*^2^(2) = 8.487, *p* = .014 comparing male control, cAG, and iAG participants.

**Figure 4:**
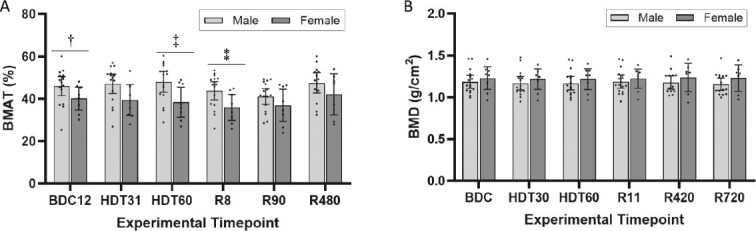
(A and B) Sex-specific modulation of vertebral bone marrow adipose tissue and BMD. (A) Mean BMAT of the lumbar spine stratified by sex for all participants combining PD, Dixon, and MRS quantitative MR data. Male had significantly larger BMAT than female participants at baseline; the difference was no longer significant 90 d and 480 d after the 60 d of bedrest. (A) Mean BMD combining vertebral levels L1-L4 stratified by sex for all participants. Whiskers correspond to 95% CIs. BMAT, bone marrow adipose tissue; PD, proton density; MRS, MR spectroscopy; BDC, baseline data collection; HDT, head-down tilt bedrest; R, reambulation; cAG, continuous artificial gravity; iAG, intermittent artificial gravity. ^†^U = 31.00, *p* = .043 comparing male and female participants. ^‡^U = 28.00, *p* = .027 comparing male and female participants. ^$\overset{*}{ *} $^U = 27.00, *p* = .023 comparing male and female participants.

**Figure 5:**
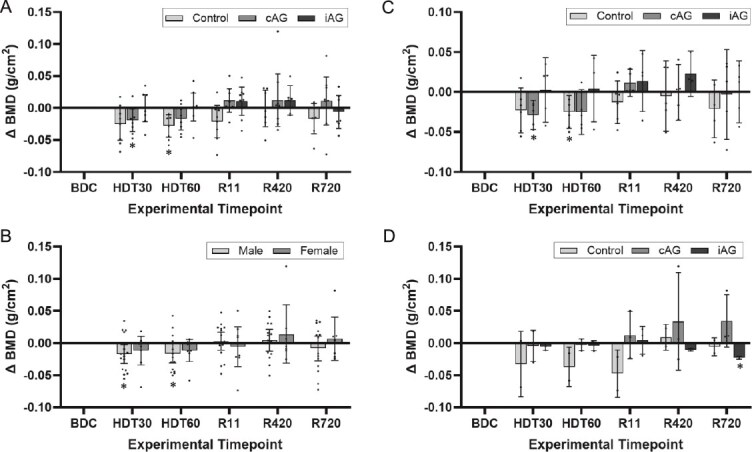
(A-D) Change in BMD stratified by intervention and sex. (A) Mean change in BMD from baseline of the lumbar spine for all participants stratified by intervention. 60 d of bedrest significantly decreased vertebral BMD in the control group. cAG at 60 d and iAG at 30 and 60 d prevented the bone loss. (B) Mean change in BMD averaging vertebral levels L1-L4 for all participants stratified by sex. The vertebral bone loss after 60 d of bedrest reached statistical significance in the male participants. Mean change in BMD of the lumbar spine stratified by intervention for male (C) and female (D) participants. cAG at 60 d and iAG at 30 and 60 d prevented the bone loss in male participants. Whiskers correspond to 95% CIs except for (D), where whiskers correspond to ±1 SD. SD is used for female participants due to the large 95% CI associated with *n* = 2, *n* = 3, and *n* = 3 for control, cAG, and iAG subgroups, respectively. BDC, baseline data collection; HDT, head-down tilt bedrest; R, reambulation; cAG, continuous artificial gravity; iAG, intermittent artificial gravity. *p < .05 compared to baseline.

### Effect of artificial gravity interventions on vertebral BMD

Participants in the control and cAG groups showed significantly decreased BMD during bedrest: participants subject to cAG had a significant decrease in BMD at HDT30, −0.019 g/cm^2^ (95% CI: −0.037 to −0.002, *t*(2.364), *p* = .036) and control participants had a significant deceased in BMD at HDT60, −0.028 g/cm^2^ (95% CI: −0.045 to −0.010, *t*(7) = 2.364, *p* = .007), compared to baseline. There were no significant differences between interventions (Figure 5A).

### Sex-based modulation of vertebral BMD

At baseline, female participant’s lumbar spine BMD was 1.23 g/cm^2^ (95% CI: 1.09 to 1.36) and male participants was 1.18 g/cm^2^ (95% CI: 1.11 to 1.26; Figure 4B). The male:female differences in vertebral BMD were not statistically significant at all study time points (Figure 4B). Bed rest significantly decreased male participant’s lumbar vertebral BMD compared to baseline; 30 d, −0.017 g/cm^2^ (95% CI: −0.032 to −0.002; *t*(15) = 2.131, *p* = .028) and 60 d, −0.016 g/cm^2^ (95% CI: −0.031 to −0.001, *t*(15) = 2.131, *p* = .037, Figure 5B). Likely limited by the low final sample size per group, the female participants’ vertebral BMD during bedrest were not significantly different than baseline. The differences between sexes were not significant.

### Sex-based response of vertebral BMD to bed rest and to artificial gravity

The male participants subjected to the iAG intervention showed no decrease in BMD during HDT bed rest, whereas BMD decreased for male participants subjected to cAG after 30 d of bedrest (−0.029 g/cm^2^; 95% CI: −0.047 to −0.010) and male control after 60 d of bed rest (−0.025 g/cm^2^; 95% CI: −0.046 to −0.004) compared to baseline (Figure 5C). There were no differences between groups. The female control participants’ vertebral BMD during bedrest were not significantly different than baseline although lacking statistical power to draw definite conclusions (Figure 5D).

### Effect of bed rest and Reambulation on vertebral BMD

At baseline the mean lumbar spine BMD for all participants was 1.20 g/cm^2^ (95% CI: 1.14 to 1.26; Figure 2B). Lumbar vertebrae BMD decreased significantly after 30 and 60 d of HDT bed rest (Figure 2B). The mean change in BMD for all participants after 60 d of HDT bed rest was −0.014 g/cm^2^ (95% CI: −0.025 to −0.004, *t*(23) = 2.815, *p* = .010, Figure 2B). BMD returned to baseline after 11 d of reambulation.

### Correlation between vertebral marrow adipose tissue and BMD

We explored the correlation between BMAT and BMD by plotting BMAT as well as change in BMAT against BMD and change in BMD, respectively (Figure S3A and B). Correlations were stratified by MR techniques (Figure S4A-C In all 5 comparisons, the direction of the correlation was inverse (Figures S3 and S4).

## Discussion

This prospective randomized controlled intervention trial provided the first evidence that AG blunted BMAT accumulation in the lumbar spine of participants during 60 d of bedrest. BMAT accumulated in controls compared to participants subjected to either AG protocols at 2 g for 30 min/d. Previously, only one mechanical countermeasure had shown effectiveness at preventing BMAT accrual during bed rest: resistive exercise with or without whole-body vibration.^6^ In addition, the male participants who received iAG showed no bone loss at 30 d nor at 60 d of bedrest. The current results support that recreating an axial force vector mechanically prevented bone and bone marrow effects of skeletal offloading at the lumbar spine in bedridden people. That both iAG and cAG mitigated BMAT increases during bedrest while only iAG mitigated BMD changes may either reveal superiority of iAG over cAG or be a limitation related to sample size.

Bone and bone marrow are mechanosensitive.^33^ Inactivity, microgravity, and hibernation were shown to lead to BMAT accumulation and loss of BMD^5^^,^^17^ To the contrary, exercise was correlated with lower BMAT content at the lumbar vertebrae.^7^^,^^34^ The mechanisms for BMAT accumulation with inactivity remain incompletely understood. Bone marrow adipocytes share the same MSC precursor as osteoblasts and adipokines modulate myeloid cells, osteocytes, and osteoclasts function.^35–37^ Increases in BMAT have been correlated with low bone density.^18–21^ Cancellous bone cultures in microgravity demonstrated that mechanical loading blunted bone resorption, whereas the same loading pattern increased bone formation in normogravity.^38^ Animal models of limb immobilization have shown hyperplasia of small adipocytes after short durations followed by adipocyte hypertrophy after long durations of immobilization.^39^^,^^40^ Spaceflight is known to decrease the BMD of astronauts and this has been reported since early missions.^25^^,^^26^ Smith et al., Gabel et al., and Liu et al. consistently measured increased bone resorption markers and decreased calcium absorption in astronauts on long duration missions.^14^^,^^41^^,^^42^ Astronauts’ bone anabolic markers were unchanged at landing compared to preflight but increased significantly 2-3 wk postflight.^41^ Clement et al. previously reported hip BMD in the same population.^30^ Trochanter in the cAG group was decreased −3.2% at R11, while FN was decreased −1.3% in the Control group compared to baseline. In our study, BMD decreased during HDT bed rest in cAG and control participants, consistent with these studies and the application of intermittent AG forces prevented BMD losses during bed rest.

### BMAT inversely correlated with BMD

The inverse function between BMAT and BMD has long been reported.^15^^,^^18–21^ The medullary space of any bone, including a vertebrae is not expansible. The finite marrow volume dictates that BMAT accumulation can only occur at the expense of the other bone marrow constituents: the trabecular bone and the hematopoietic tissue. In this study, the inverse relationship between BMAT and BMD was identified across the bedrest and reambulation phases. One possible mechanism is the adipose switch: when subjected to mechanical forces, pluripotential mesenchymal stem cells (MSC) differentiate preferentially to bone and in the absence of forces, MSCs differentiate to fat.^43^ This model requires the change in forces to apply over long enough periods of time for adipocytes differentiation and proliferation to quantitatively influence the MR signal. Sixty days of bedrest appears a long enough duration to achieve adipose differentiation and proliferation/hypertrophy. Additionally, the BMAT accrual with inactivity was accompanied in this study and in others by bone loss.^5^^,^^6^^,^^26^^,^^44^

The reambulation period was different. A 3.86% reduction in vertebral BMAT between the sixtieth day of bedrest and the eighth day of reambulation (only 8 d) appeared too short to invoke a MSC differentiation switch from fat to bone. Switching off the MSC adipose differentiation would not so rapidly decrease the fat signal. These findings reproduce results of a previous bedrest study that measured a 10% decrease in BMAT signal after 30 d of reambulation^12^ and astronauts returning from ISS with a 4.2% decrease in BMAT after 41 d of reambulation.^45^ A more rapid and responsive explanation must be considered. A mechanism based on bone marrow energetics may alternatively explain the rapid changes.^46^ Reambulation after prolonged inactivity stimulates bone anabolism to overcome immobilization osteopenia.^47^ Reambulation also stimulates erythropoiesis to overcome anemia of immobility.^48^ Anemia of immobility has reproducibly occurred in long-duration bedrest studies and after returning from space (space anemia).^12^^,^^14^^,^^48^ Both bone marrow functions, bone anabolism and erythropoiesis, are energy-intensive and their preferential source of energy may be the locally and readily available fatty acids in the bone marrow.^35^^,^^46^ Intensive consumption would lead to rapid depletion of fatty acid stores in bone marrow adipocytes and/or osteocytes, resulting in the decreased BMAT MR signal at reambulation.^49^ The bone marrow energetics mechanism, like the adipose switch mechanism would maintain an inverse relationship between BMAT and BMD, as seen in this study. The local bone marrow energetics mechanism may contribute to explain BMAT decrease in hypermetabolic bone metastases and BMAT increase after treatment, BMAT increase after radiation which decreases marrow cellular metabolic activity.^36^^,^^37^^,^^50^ This may also explain why subgroups with limited BMAT accumulation in this study (women, men with AG interventions) still underwent BMAT reconversion at reambulation.

### Sex-based BMAT and BMD modulation

Female participants had lower BMAT than male participants at baseline (Figure 4A). This is consistent with studies that have demonstrated sex-specific differences in BMAT content for age matched subjects.^9–11^^,^^16^ The M:F physiological difference in BMAT was no longer significant 480 d after bedrest mainly owed to an increased BMAT in female participants. These results are consistent with persistent increases in BMAT reported both in female participants after bedrest and in female astronauts one year after long-duration exposure to space.^5^^,^^14^ These increases long after bedrest or space exposure were not seen in male participants to bedrest studies or male astronauts.^5^^,^^14^ The mechanisms modulating BMAT are often separately considered for women and men.^51–53^ Microgravity environments may alter estrogen levels.^51^ Estrogen-deficient states, such as functional hypothalamic amenorrhea and anorexia, elevate BMAT compared to healthy controls.^52^^,^^53^ When treated with transdermal estradiol therapy, both populations decreased their lumbar BMAT. Estrogen levels were not measured in the current study where contraceptives were not allowed, and we found no literature supporting changes in estrogen levels with inactivity in women. Since the literature direly lacks studies with sufficiently powered female representation, the current results, although aligned with prior studies, may only become definitive when sample size is suitably expanded.

While the clinical application of the AG protocols delivered in this study is currently neither widely available nor tested in clinical populations, the proof of principle constitutes a major step forward. The AG intervention could be tested in specific subpopulations unable to load bear or to be physically active and therefore exposed to acute or chronic BMAT accumulation or loss in bone mass. These include athletes temporarily unable to train due to injuries, people with acute paralysis, patients receiving acute bone-depleting medication such as high-dose systemic corticosteroids, or chronic wheelchair users, including para-athletes, and those confined to bed. Astronauts looking to prevent bone loss during prolonged exposure to space may benefit from AG applications. Whether AG could be used prospectively for bone accretion in populations with lower bone stock remains to be studied. The benefits of delivering AG to these populations can drive technological advancements.

There are several limitations associated with the presented work. While this study design introduced an obvious statistical bias by recruiting 8 female participants for 16 male participants, the sex-differences in BMAT are worth noting given their systematic occurrence and similar reports in the literature.^43^ Increased intervertebral disc height that may occur with antiorthostatic bedrest could lower BMD readings in all three groups.^54^ The effectiveness of the countermeasures will benefit from confirmatory studies with larger sample sizes and equal sex-distribution.

## Conclusion

Artificial gravity applied for 30 min per day prevented vertebral BMAT accumulation and bone loss during prolonged bedrest. Bone marrow adipose tissue and BMD in the same vertebrae were inversely modulated. Artificial gravity may be clinically applicable to populations at risk of acute or chronic bone loss.

## Supplementary Material

fig_S1_zjaf119

fig_S2_zjaf119

fig_S3_zjaf119

fig_S4_zjaf119

Supplementary_Table_1_zjaf119

## Data Availability

The data underlying this article were provided by the European Space Agency. Data will be shared on request to the corresponding author with permission of the European Space Agency.
