# Supplementary figures and images for: Artificial gravity protects bone and prevents bone marrow adipose tissue accumulation in humans during 60 d of bed rest
Source: J Bone Miner Res. 2025 Aug 28;40(11):1218–27. doi: 10.1093/jbmr/zjaf119 (PMC12578298; doi:10.1093/jbmr/zjaf119)

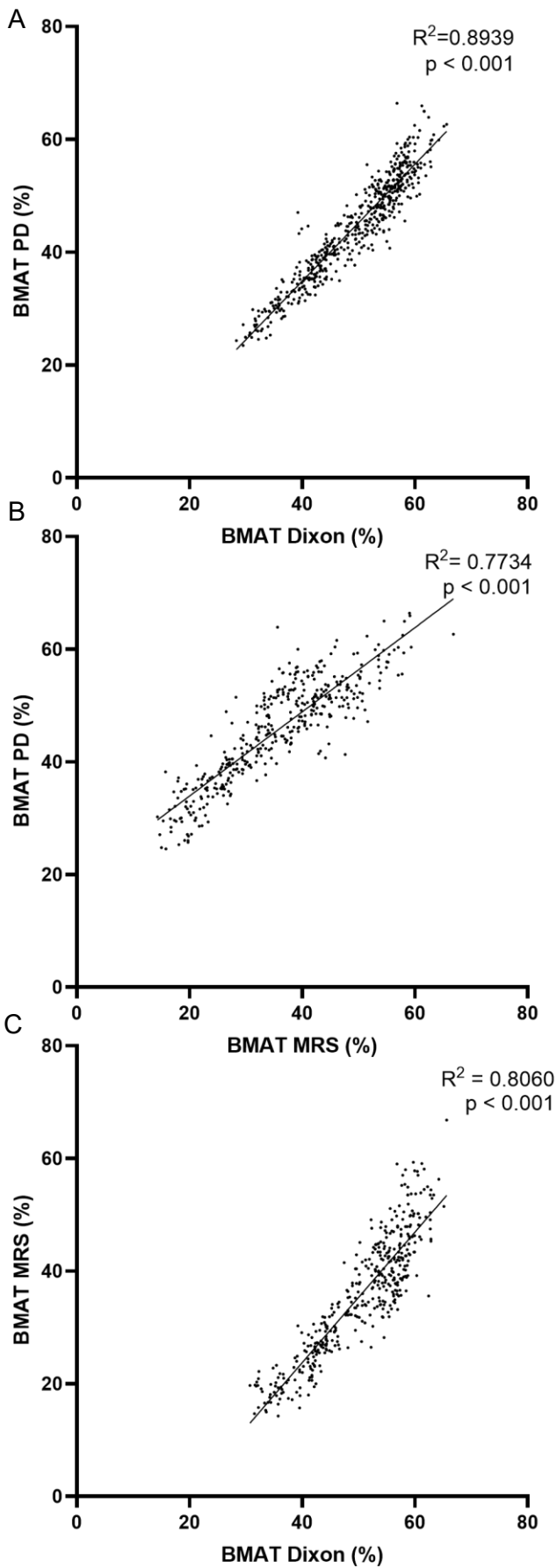

Supplement: fig_S1_zjaf119 [file fig_s1_zjaf119.pdf]

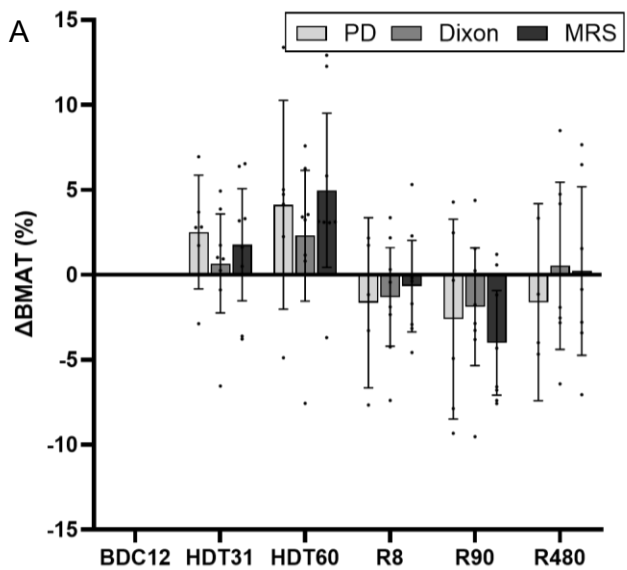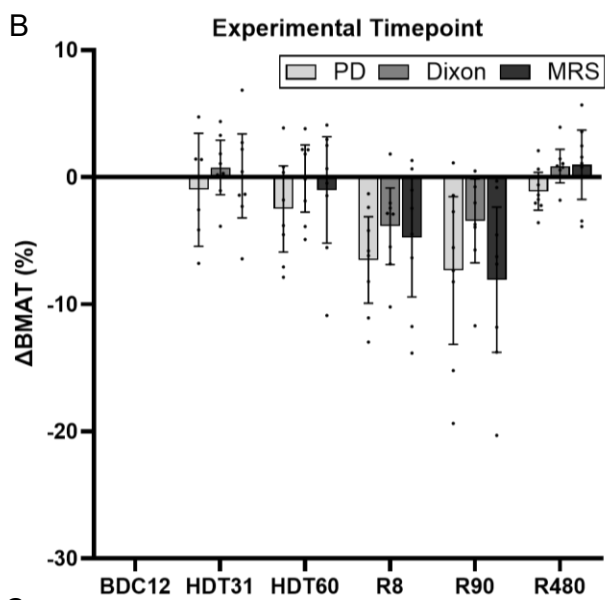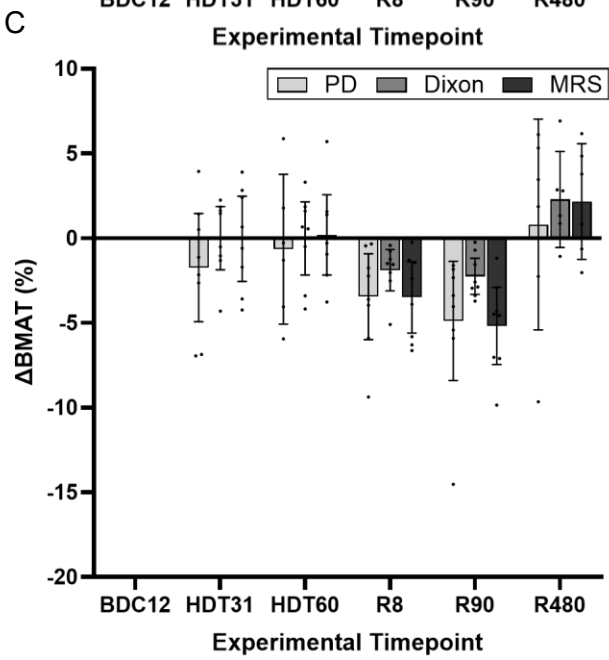

Supplement: fig_S2_zjaf119 [file fig_s2_zjaf119.pdf]

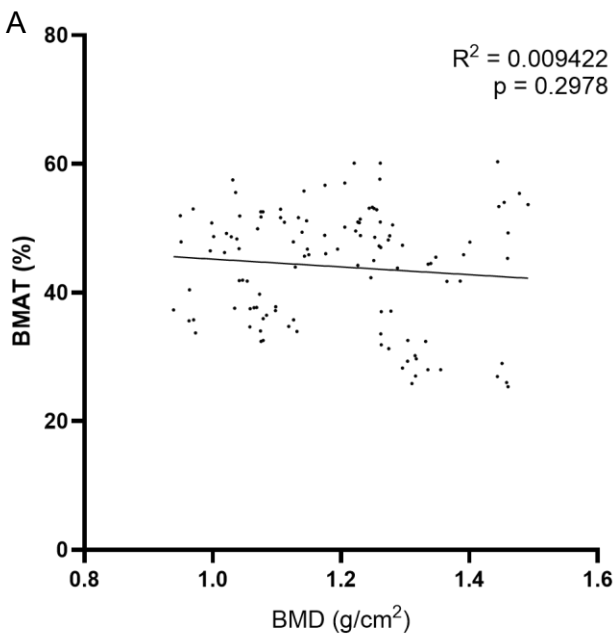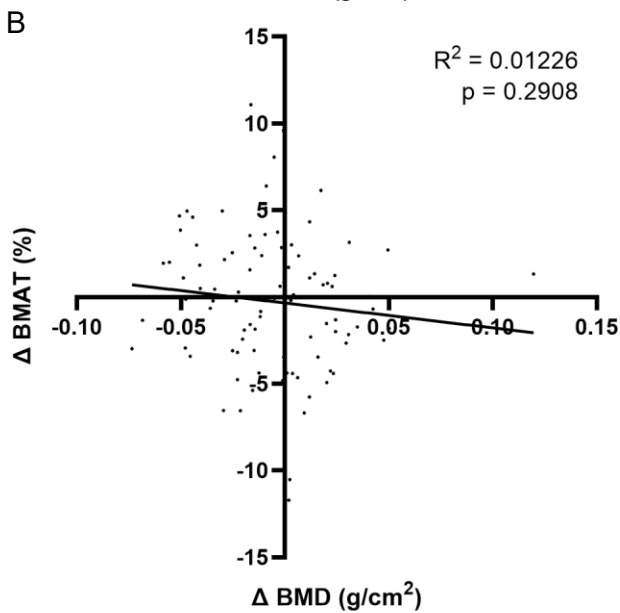

Supplement: fig_S3_zjaf119 [file fig_s3_zjaf119.pdf]

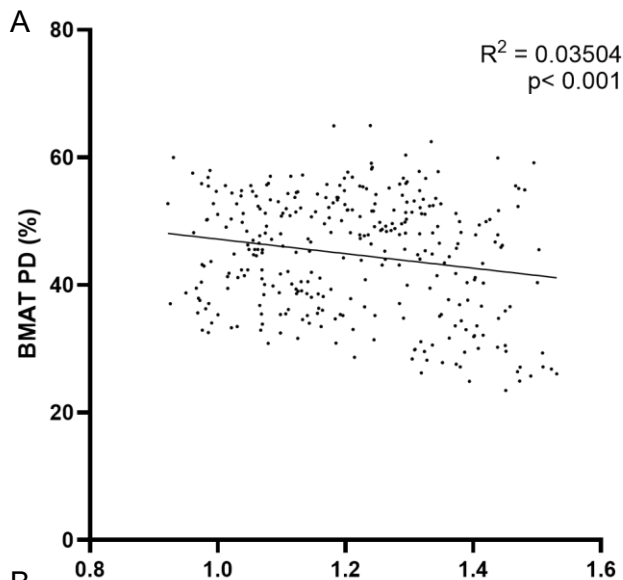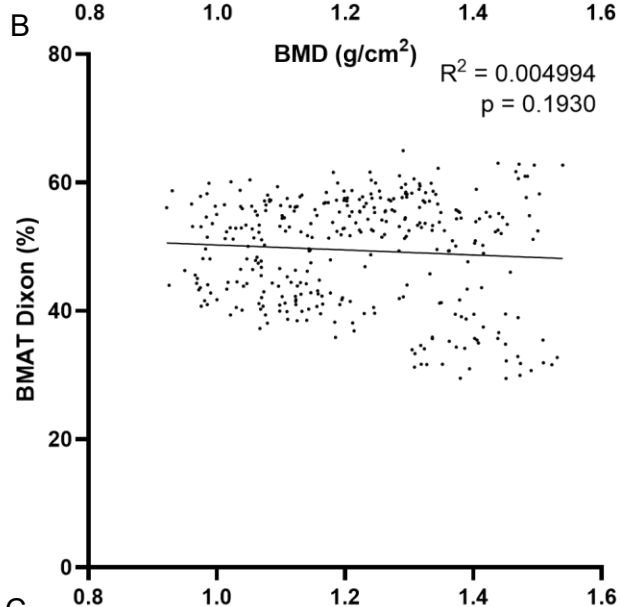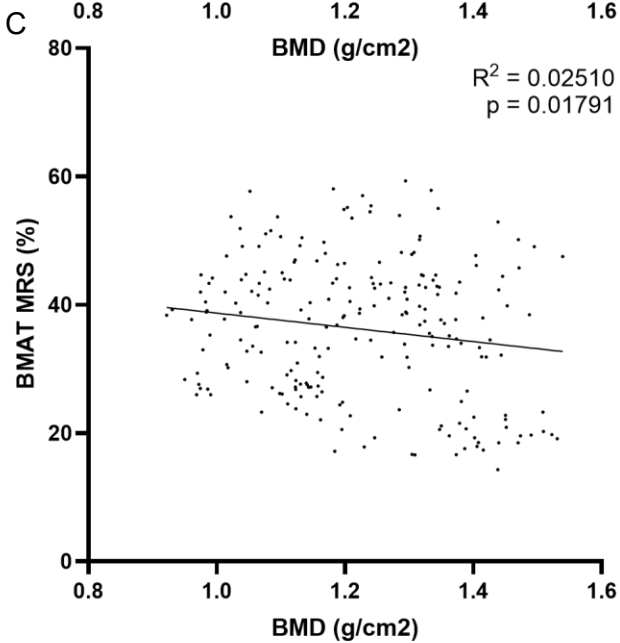

Supplement: fig_S4_zjaf119 [file fig_s4_zjaf119.pdf]
