# Supplementary material for: Artificial gravity protects bone and prevents bone marrow adipose tissue accumulation in humans during 60 d of bed rest
Source: J Bone Miner Res. 2025 Aug 28;40(11):1218–27. doi: 10.1093/jbmr/zjaf119 (PMC12578298; doi:10.1093/jbmr/zjaf119)
Supplement: Supplementary_Table_1_zjaf119 [file supplementary_table_1_zjaf119.docx]

Supplementary Table 1: Effect of 60 days of HDT bedrest on lumbar spine BMAT for all 24 participants using PD, Dixon, and MRS techniques.

|  | Bone marrow adipose tissue (%) | | | |
| --- | --- | --- | --- | --- |
| Time | All Techniques  Mean (95% CI) | PD  Mean (95% CI) | Dixon  Mean (95% CI) | MRS  Mean (95% CI) |
| BDC12 | 44.1 (40.6-47.5) | 45.9 (42.1 – 49.6) | 49.9 (46.7 – 53.1) | 35.1 (30.1 – 40.1) |
| HDT31 | 44.5 (40.6 – 48.4) | 46.1 (42.0 – 50.2) | 50.4 (46.8 – 54.0) | 37.1 (32.5 – 41.7) |
| HDT60 | 44.8 (40.6 – 49.0) | 47.1 (42.9 – 51.3) | 50.6 (46.9 – 54.4) | 37.9 (33.0 – 42.8) |
| R8 | 41.2 (37.6 – 44.8)* | 41.6 (38.0 – 45.2)* | 47.6 (44.1 – 51.0)* | 33.6 (29.4 – 37.7)* |
| R90 | 39.7 (36.5 – 43.0)* | 41.0 (37.7 – 44.4)* | 47.4 (44.1 – 50.6)* | 30.8 (27.3 – 34.2)* |
| R480 | 45.7 (41.5 – 49.9) | 46.8 (42.5 – 51.1) | 51.9 (48.1 – 55.6) | 38.2 (33.3 – 43.2) |

*Significant difference from BDC12, *p*<0.05.
